# Looking towards the sweet, sweet future: a political economy analysis of sugar and nutrition policy in Indonesia

**DOI:** 10.1017/S1368980025100566

**Published:** 2025-06-17

**Authors:** Ellen Johnson, Hafizah Jusril, Liza Pratiwi, Suci Trisnasari, Anne Marie Thow, Cut Novianti Rachmi

**Affiliations:** 1 Leeder Centre for Health Policy, Economics and Data, Sydney School of Public Health, The University of Sydney, Sydney, NSW, Australia; 2 Reconstra Utama Integra, Setia Budi Building 2, Suite 207C; HR Rasuna Said Kav 62, Jakarta Pusat 12920, Indonesia; 3 STOP TB Partnership Indonesia, Gedung Medco 1, Lt 2. Jl. Ampera Raya 18-20 Cilandak Timur, Pasar Minggu, Jakarta Selatan 12560, Indonesia

**Keywords:** Political economy, Nutrition policy, Sugar, Economic policy, Food security

## Abstract

**Objective:**

To identify politico-economic factors relating to policy surrounding the production, processing and trade of sugar in Indonesia and identify strategies to support improved integration of national nutrition and food security priorities with respect to sugar.

**Design:**

This study was a qualitative policy analysis, informed by political economy and power analysis approaches and drawing on both documentary policy data and interviews.

**Setting:**

Indonesia.

**Participants:**

Interviewees from various national and sub-national government and non-government sectors, with expertise in health and food safety (*n* 7), finance and economics (*n* 2), trade and industry (*n* 3) and others (*n* 4).

**Results:**

Sugar was articulated as a policy priority in three distinct ways: (1) sugar as an economic good; (2) sugar in relation to health and (3) sugar as a commodity for food security. High political priority was given to national economic development, as well as concerns relating to farmer rights and welfare. Nutrition priorities and objectives to reduce sugar consumption were addressed in health policies; however, they were not reflected in production and economic policies promoting sugar.

**Conclusions:**

Creating opportunities to diversify agricultural production and ensuring a just transition to protect the livelihoods of sugar farmers in Indonesia will be crucial in enabling the achievement of nutrition priorities to reduce sugar consumption.

Non-communicable diseases (NCD) account for 74 % of all deaths globally, with 77 % of all NCD deaths occurring in low- and middle-income countries^(1)^. Unhealthy diets – particularly those high in free sugars, fat and Na – are associated with an increased risk of NCD morbidity and mortality. Excess sugar consumption in the diet is of particular concern, with the WHO guidelines for sugar intake for adults and children highlighting the association between high consumption levels of free sugars, obesity and increased risk of NCD^(2,3)^. ‘Free sugars’ are sugars ‘…added to foods and beverages by the manufacturer, cook or consumer and sugars naturally present in honey, syrups, fruit juices and free juice concentrate’^(2)^. The WHO guidelines strongly recommend reducing the intake of free sugars to less than 10 % of total energy intake and conditionally recommend a further reduction to below 5 % of total energy intake^(2)^.

Policy action will be an essential component of government efforts to reduce sugar consumption, addressing both supply and demand for sugar. However, there is a tension globally between efforts to reduce sugar consumption, and sugar as an important economic commodity for many countries. The sugar industry – including the production, trade and distribution of sugar – comprises a large economic sector in many countries including Brazil, India, Australia and Indonesia^(4)^. This makes sugar policy a political issue, as economists within industry and governments raise concerns about adverse effects on the economic livelihoods of sugar farmers, processors and small businesses^(5)^. Sugar farmers are often low-income^(6)^ and although shifting away from sugar production can offer environmental and livelihood benefits^(5,7)^, this can present challenges. Previous analyses indicate the influence of industry on creating resistance to the adoption and implementation of impactful nutrition policy^(8–11)^, that aims to address health-related issues linked with unhealthy diets. In Indonesia, the sugar and associated industries have strongly opposed policy action on sugar, including vocal resistance to a proposal by the Ministry of Finance to implement a sugar-sweetened beverage tax^(12)^.

Efforts by public health nutritionists to reduce sugar consumption thus need to take into account the need, in many countries, to also address supply-side factors that are promoting sugar production. This includes a need to re-orient structural incentives that promote sugar production, processing and trade in sectors including agriculture, commerce and trade. However, there has been limited integrated analysis of the varied policy agendas relating to sugar, and few efforts to inform public health nutritionists of how they could effectively encourage policy change across these sectors. In this study, we examined the policy context related to sugar across sectors in Indonesia, a country with a significant domestic sugar sector, and identified opportunities to resolve policy tensions related to reducing sugar consumption. These lessons will provide important insights globally regarding strengthening policies to reduce sugar consumption.

## Indonesia

The double burden of malnutrition is a prevailing health challenge in many low- and middle-income countries, including Indonesia. Slow progress has been made in reducing stunting, with 21·5 % of children under 5 years being stunted while 4·2 % of children recorded were overweight in 2023^(13)^. The prevalence of anaemia among women of reproductive age is still high, with 29 % of women aged 15–49 years affected^(14)^. Additionally, changes in diet in Indonesia, such as the increased consumption of processed foods – which are typically high in fat, sugar and salt – are contributing to increased prevalence of overweight and obesity and diet-related NCD^(3,15)^. Per capita consumption of sugar in Indonesia increased by 22 % between 2009 and 2017^(16)^. Indonesians are the third largest consumer of sugar-sweetened beverages in South-East Asia, with two out of three people aged 5–19 years consuming one or more servings of sugar-sweetened beverages per day^(17,18)^. In 2018, 14·4 % and 23·4 % of people in Indonesia were overweight and obese respectively^(13)^, and in 2016, the economic cost of overweight and obesity-related illness was estimated at 1·51 trillion rupiah (US$116 million) in direct medical costs and 368·3 trillion rupiah (US$28·3 million) in lost productivity^(12)^. In 2019, Indonesia had close to 10 million adults living with type 2 diabetes, which is one of the highest rates globally^(19)^. Nearly 19 % of Indonesians aged 15 years and above are pre-diabetic^(13)^.

Given the nature of NCD risk factors in Indonesia, policies to reduce sugar consumption are a priority. Encouraging the replacement of sugar consumption with nutrient-dense foods and beverages will also support reductions in malnutrition. However, sugar is a major economic sector in Indonesia, and the central government classifies sugar as one of seven strategic food commodities^(20)^. In 2017, the Ministry of Trade classified sugar as one of ‘sembako’ – one of the nine products deemed necessities for daily living^(21)^. The value of Indonesia’s domestic sugar industry is estimated at $IDR 25 trillion ($USD 1·74 billion), accounting for 0·2 % of total GDP^(22,23)^. Indonesia’s sugar industry includes 746 037 farmers and 267 931 labourers^(24)^. The large labour force and value of the sugar industry underpin its importance to the national economy and rural livelihoods. However, domestic production can no longer meet demand, and 52 % of sugar is imported from other countries^(16)^. In this context, the government of Indonesia is undergoing a reform of the domestic sugar industry, in a bid to bolster domestic production, trade and distribution.

As a result, there are policy tensions regarding sugar between government sectors focussed on nutrition and health priorities, economic priorities and food security priorities. Understanding politico-economic and power dynamics that underpin the agenda and priorities of different actors regarding sugar, including a broader analysis of the changing historical context of agricultural and trade policies in Indonesia, can help inform policy that both supports the economy and protects health in the future. In particular, political economy and power analysis can identify opportunities for improved policy coherence and support health policymakers engage across sectors, to achieve national nutrition and food security priorities^(25,26)^. The aim of this study was to analyse the political economy of sugar-related policy in Indonesia and identify strategies to improve the integration of national nutrition and food security priorities in this policy space.

## Methods

### Study design and framework

This study was a qualitative political economy and power analysis, drawing on documentary policy and interview data. Acosta and Pettit^(27)^ outline four key elements to their approach to political economy and power analysis: (1) *Formal and visible structures and institutions (norms and ‘rules of the game’)*. This element includes formal power ‘…the visible, recognised structures of power that are part of the way in which societies work…’^(27)^. Formal power often shapes laws and rules (i.e. authoritative power) that define what is acceptable or not acceptable in a given space. (*2) Informal and invisible structures (beliefs, narratives, and discourses)*. This element includes informal or ‘invisible’ power, which is recognised through social norms, discourses and cultural practices that shape everyday life. Informal power often takes shape as discursive power, which shapes and amplifies specific ideas, narratives and beliefs. *(3) Actors, interests and strategies that influence policy*. This includes identifying the relevant actors that have influence on policy processes and establishing their powers, roles and responsibilities. *(4) Processes of cooperation and contestation* include motivations and influences on actor cooperation, building on the articulation of the first three elements on the formal and informal structures, and the interests of actors. Using a combined political economy and power analysis approach helps make sense of the power relations and political dynamics in the formulation, adoption, implementation and evaluation of policies.

### Data collection

#### Policy documents

The co-authors conducted a targeted search between October 2021 and July 2022 of government websites to identify national-level policies (acts, legislation, policy, action plans and technical guidelines) relating to the production, distribution and trade of sugar, as part of a wider search for policies relevant to health, NCD, nutrition and food security. Search terms included Bahasa Indonesia equivalents for ‘sugar’, ‘nutrition’, ‘health’, ‘trade’, ‘agriculture’ and ‘production’. A total of forty-eight policy documents were included. Policies were deemed relevant to sugar if they contained explicit or implicit (e.g. health policies containing general recommendations on healthy diets) goals, objectives or activities related to the production, processing, distribution, trade and consumption of sugar. Policies were translated from Bahasa Indonesia to English using Google Translate and checked by Bahasa Indonesia-speaking co-authors. Policy documents were from multiple sectors including health and food safety (*n* 7), agriculture (*n* 7), industry and trade (*n* 13), finance and economics (*n* 9), national development (*n* 1) and other (*n* 11). A matrix for data extraction was created in Microsoft Excel™. Policy content was extracted by the first author, relating to the responsible ministry or department, policy objectives, priorities and actions relevant to sugar and mechanisms and approaches to policy coordination.

#### Interviews

A total of sixteen in-depth interviews were conducted by co-authors with interviewees from various national and sub-national government and non-government sectors: health and food safety (*n* 7), finance and economics (*n* 2), trade and industry (*n* 3) and others (*n* 4). Interviewees were identified through policy mapping and then through snowball sampling. Interviewees were recruited through formal letters of invitation. A semi-structured interview guide was developed in English and translated to Bahasa Indonesia for the purpose of conducting the interviews. (with back-translation for verification) The interview guide included questions on understanding nutrition policy (including problems and priorities), stakeholders and ministries involved in sugar policy, government and ministry priorities with respect to sugar policy and challenges in implementing policy relevant to sugar and domestic sugar production, trade and distribution. Interview data relevant to this analysis were translated from Bahasa Indonesia to English for the analysis by the research team.

### Data analysis

Informed by Acosta and Pettit’s approach to political economy and power analysis, we drew on the documentary policy data to identify: formal and visible structures (what are the legal frameworks, norms and regulations defining existing policy), informal and invisible structures (beliefs and narratives surround sugar policy, what are the predominant national identities related to sugar) and the main actors involved in policy and their interests and the processes of cooperation and contestation of actors (what are the actors motivations to cooperate). Data analysis was carried out manually in Excel. Documentary policy data were coded deductively to the codes above initially by the first author and checked by a co-author. Patterns in the policy data were marked with codes, and similar codes were grouped into broader themes. Based on this analysis, the following politico-economic factors had a strong influence on policy surrounding the production, processing and trade of sugar in Indonesia: framing of sugar as an economic good; framing of sugar as a commodity for food security and framing of sugar in relation to health.

Informed by the theoretical framework and study aims, interview data were coded deductively, by the first author and checked by a co-author. Data analysis was carried out manually in Excel. Patterns in the interview data were marked with codes, and the data were thematically analysed within these codes, with a focus on norms, actors, interests, power and policy cooperation and contestation. Similar codes were grouped into broader themes and categorised as highlighted in Table 1. An integrated analysis was then conducted, with the findings from the documentary policy data triangulated with those from the interviews. Coded interview data were compared with similarities in the emerging themes from the policy documents to strengthen the credibility and validity of the analysis. Based on this analysis, results are organised around the following key findings: incoherences between ministries aiming to reduce sugar consumption and ministries promoting sugar consumption for food security; tensions between policies reducing sugar consumption whilst actively promoting sugar production and processing and alignment between ministries framing sugar as an economic good and ministries framing sugar as a commodity for food security.

**Table 1.** Interview coding

Acosta and Pettit framework	Codes	Themes	Key findings
Formal and visible structures and institutions (norms and ‘rules of the game’)	Government policies that relate to sugar; influences on sugar policy (people, institutions, companies and contextual factors); broad context for sugar policy	History of sugar policy (including contextual factors) Competing priorities (e.g. malnutrition and child/maternal health) Barriers to sugar policy formation (for health) MSMEs^*^, farmers, business sector – trade-offs and who ‘loses out’ with stricter sugar policy Culture Competing priorities	Incoherence between ministries aiming to reduce sugar consumption and ministries promoting sugar consumption for food security Tensions between policies reducing sugar consumption, whilst actively promoting sugar production and processing Alignment between ministries framing sugar as an economic good, and ministries framing sugar as a commodity for food security
Informal and invisible structures	Policy ‘problem’ of sugar; change in government priority or interest related to sugar industry	Trends in consumption (including social norms/‘trending and cool’)	
Actors, interests, and strategies	Government ministries relevant to sugar policy; private sector actors; NGO’s or international organisations	Who regulates sugar Policy priority tensions Framing and beliefs around sugar	
Process of cooperation and contestation	Coordination or implementation of sugar policies; opportunities to improve sugar policy	Policy gaps Coordination and cross-sectoral engagement Barriers to sugar policy formation (for health) MSMEs, farmers, business sector – trade-offs and who ‘loses out’ with stricter sugar policy Culture Competing priorities	

* Micro-, small- and medium-sized enterprises.

## Results

### Current policy landscape and priorities

**Figure 1.**
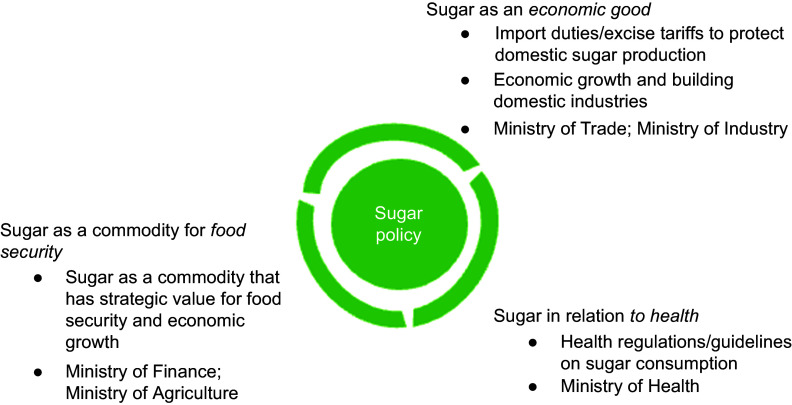
Policy priorities and framing of sugar policy in Indonesia.

This analysis identified three distinct types of policy priorities related to sugar in Indonesia: (1) sugar as an economic good; (2) sugar in relation to health and (3) sugar as a commodity for food security. These different priorities are accompanied by specific policy activities and framings (Figure 1).

#### Sugar in relation to health

In the context of rising rates of NCD such as diabetes, the Ministry of Health has promulgated policies that prioritise reducing sugar consumption to reduce disease burden. Health sector policies link unhealthy diets (e.g. high in sugar) to the emergence and prevalence of NCD and provide specific recommendations regarding limiting sugar intake, as well as broader healthy diet recommendations. These policies contextualise the burden of NCD in Indonesia within the context of unhealthy diets and sub-optimal nutrition. Whilst the Ministry of Health is the primary ministry creating policies encompassing guidelines related to sugar consumption for health, other areas of government have relevant policies (Table 2). Finance sector policies relate to the potential to expand excise duties, including on high-sugar foods, and national laws have general mandates for health, including the acknowledgement that health is a human right that is the responsibility of both the Government and community.

**Table 2.** Examples of policies relevant to reducing sugar consumption for health

Policy	Year	Relevant content
Minister of Health Regulation No. 21/2020 on the Strategic Plan of the Minister of Health, 2020–2024	2020	Recommendations on sugar consumption Recommends consuming no more than four tablespoons of sugar per day^(41)^ ‘Unhealthy eating patterns contribute to PTM (Penyakit Tidak Menular – Non Communicable Diseases). Foods high in sugar, salt and fat and low in fibre are contributors to PTM.’^(41)^
Minister of Finance Regulation No. 77/PMK.01/2020 on the Strategic Plan of the Ministry of Finance, 2020–2024	2020	Recommendations on expanding excise duties ‘…expanding excise duties on high-risk food products on health and regulation of food products with sugar, salt and fat content.’^(42)^
Minister of Health Regulation No. 14/2014 on Guidelines for Balanced Nutrition	2014	Broad dietary recommendations Limit consumption of sweet food and drinks^(43)^ Reduce the use of sugar, both in various drinks (tea, coffee, milk, juices and other sugar drinks, as well as in various foods, snacks and when adding to dishes^(43)^ Replace sweet desserts with fruits and/or vegetables^(43)^
Law No. 36/2009 on Health	2009	Recognises health as a human right ‘Health is a human right…and must be realised in accordance with the ideals of the Indonesian nation…’^(44)^ ‘Health is a state both physically, mentally, spiritually and socially which enables everyone to live productively, socially, and economically…’^(44)^

#### Sugar as an economic good

Sugar was identified as a key economic commodity for Indonesia, primarily through Ministry of Industry and Ministry of Trade policies (Table 3). Ministry of Industry policies provide financial relief to sugar factories in the form of subsidies/discounts for sugar factory machinery and equipment. Ministry of Industry also supports sugar as an economic good through the establishment of a team dedicated to providing direction and coordination of the Sugar Industry Revitalization Action Plan. Trade sector policies included price control for commodities that are part of BULOG – a statutory corporation in Indonesia formed to regulate food distribution and price control of certain key commodities. This policy enables changes to the reference price for purchases by farmers and sale of commodities for consumers, to guarantee the availability, stability and price of rice, corn, soybeans, sugar, oil, fried meat, shallots, beef and eggs.

**Table 3.** Examples of policies relevant to sugar as an economic good

Policy	Year	Relevant content
Minister of Trade Regulation No. 27/M-DAG/PER/5/2017 on Determination of Reference Rates of Purchasing at Purchasing at Farmers and Reference Rates in Consumer Sales	2017	Controls the price of sugar through BULOG (statutory corporation for food distribution and price control) Changes reference price of purchase from farmers, and sale to consumers, in order to guarantee availability and price stability of sugar^(21)^
Minister of Industry Regulation No. 50/M-IND/ PER/3/2012 on Sugar Industry Revitalization Program Through Restructuring of Sugar Factory Machinery and/or Equipment	2012	Supporting Sugar Industry Revitalisation program Purpose is to increase production capacity and efficiency, and national sugar quality^(45)^ Support is carried out in the form of providing financial relief for the purchasing of sugar factory machinery/equipment^(45)^
Minister of Industry Regulation No. 12/M-IND/PER/1/2010 on Implementation Team of the Sugar Industry Revitalization Action Plan	2010	Establishment of the team that evaluates the Sugar Industry Revitalisation program The team is responsible for providing direction and review of the Sugar Industry Revitalisation program^(46)^ Team is composed of Ministers from Industry, Agriculture, Trade, Economic Affairs and Forestry Planning^(46)^
Minister of Industry Regulation No. 91/M-IND/PER/11/2008 on Sugar Factory Machinery/ Equipment Restructuring Program	2008	Support for sugar self-sufficiency Support for sugar industry through investing in sugar factory machinery and equipment^(47)^

#### Sugar as a commodity for food security

The Constitution of the Republic of Indonesia implicitly acknowledges food security, through broader constitutional rights such Article 28C ‘Every person shall have the right to develop him/herself through the fulfilment of his/her basic needs…’^(28)^ and Article 28H ‘…the right to live in physical and spiritual prosperity…’^(28)^. Sugar is one of seven main food commodities listed, alongside rice, maize, soybeans, poultry, chili and beef, set out in the Government’s national strategic food production targets, to support food security^(29)^. Agriculture sector policies promote increased sugar production as a driving force of national development, including through establishing working relationships between agencies in charge of agriculture at the central, provincial, district and sub-district levels (Table 4). As sugar is one of the commodities set out in the national strategic food production targets for food security, these policies are intended to improve coordination and synergy of programmes/activities between these agencies to support the increase in national food production.

**Table 4.** Examples of policies relevant to sugar as a commodity for food security

Policy	Year	Relevant content
Minister of Agriculture Decree No. 259/Kpts/RC.020/M/05/ 2020 on The Strategic Plan of the Ministry of Agriculture, 2020–2024	2020	Promotion of sugar production for national development ‘…the Ministry of Agriculture has implemented strategies to reposition agriculture as a motor driving force of national development, including…increased sugar production.’^(29)^
Minister of Agriculture Regulation No. 131/PERMENTAN/OT.140/12/2024 on Mechanisms and working relationships between agencies in charge of agriculture in support of increasing national strategic food production	2014	Establishment of mechanisms and working relationships Purpose is to improve coordination between agencies in charge of agriculture, to support increasing national strategic food production, in order to address food security^(48)^
Minister of Agriculture Regulation No. 65/PERMENTAN/OT.140/12/ 2010 on Minimum Service Standards in the Field of Provincial and District/City Food Security	2010	Increase diversity of consumption and food security National Hope Food Pattern includes sugar as a recommended food group that is consumed and contributes towards an ideal diet for food security^(49)^
Presidential Decree No. 57/2004 on the Stipulation of Sugar as Goods Under Supervision	2004	Regulating sugar trade for food security ‘…sugar is a commodity that has strategic value for food security and increased economic growth…so that domestic sugar trade…needs to be monitored’^(50)^

### Politico-economic dynamics of sugar policy

The analysis identified three distinct politico-economic dynamics characterising sugar policy in Indonesia. First, there was an evident tension between ministries prioritising reductions in sugar consumption (primarily Ministry of Health) and ministries promoting sugar consumption for food security. Second, there was a lack of policy coherence between policy priorities and activities focused on reducing sugar consumption for health and those actively promoting sugar production and processing. Third, there was a coherence between ministries framing sugar as an economic good and ministries framing sugar as a commodity for food security, which was explicitly linked to addressing undernutrition but was at odds with NCD prevention policies.

#### Incoherence between ministries aiming to reduce sugar consumption and ministries promoting sugar consumption for food security

The analysis identified an incoherence between ministries aiming to reduce sugar consumption and ministries actively promoting sugar consumption for food security, including addressing undernutrition. Agriculture sector policies reflected food security initiatives aimed at reducing the burden of malnutrition and stunting focused on increasing calories (i.e. increasing food production). Interviewees indicated an underlying perception of undernutrition being much more important than NCD prevention – for example, *‘Our problem is facing undernutrition, malnutrition and stunting, and the deficiency is much more severe, compared to the problem of over nutrition’* (Government_federal_#1). The Government’s national strategic food production targets to support food security are reflected in The Strategic Plan of the Ministry of Agriculture, 2020–2024 ‘…the Ministry of Agriculture has implemented strategies to reposition agriculture as a motor driving force of national development, including…increased sugar production’^(29)^. Ministries had clear roles and responsibilities for developing policies related to the production and distribution of sugar and were aware of the role they played and who governed this space – *‘Sugar policy is with the Ministry of Trade and the Ministry of Agriculture. The Ministry of Agriculture is concerned with the crops (sugarcane), the Ministry of Trade is related with the import’* (Government_trade and industry_#1).

Health sector policies provided a range of explicit and implicit guidelines regarding sugar consumption and healthy diets. These were primarily focused on the impact of unhealthy diets (e.g. high in sugar) on the prevalence of NCD and communication regarding healthy diets. Interviewees commented on the culture surrounding sugar as a barrier to regulating sugar for health. Particularly, interviewees noted that there were difficulties in translating health messages around excess sugar consumption to the wider community due to the consumption of sugar and sugary products being so ingrained in diet culture. For example – *‘Behaviour, yes, behaviour change is necessary; our society must be self-aware that [sugar] not good, and maybe they shouldn’t consume it. But in reality, ‘I want to eat [sugar] to be cool’ …there is still a trend like that’* (Government_provincial_health_#1).

#### Tension between policies reducing sugar consumption, whilst actively promoting sugar production and processing

Alongside this incoherence, a clear theme that emerged from the policy and interview data was a tension between policies aimed at reducing sugar consumption to address the rising prevalence of NCD and policies that actively work to increase domestic sugar production and processing. This was evident within the current and historical context of sugar policy in Indonesia and competing policy priorities. Historically, Indonesia was self-sufficient in sugar production, and policies reflected this by restricting imports of sugar. However, in recent years, Indonesia has faced low domestic sugar production, and high domestic demand for sugar, particularly for industry use. This shift has been accompanied by the emergence of policies that aim to increase sugar production and revitalise the sugar industry (such as investment in sugar factories), as well as policies that regulate sugar imports in order to control the availability and price stability of sugar.

*‘…So for sugar, the government treats it the same as rice, trying to meet domestic needs through supply in the country, both from the sugarcane plantations, the factory itself and for the distribution of imports and the government’s budget…’* (Government_finance_#1).

Policies relevant to the strengthening of sugar production and processing were primarily governed by Ministry of Industry, Ministry of Trade, and Ministry of Finance. These policies included price stabilisation, subsidies for sugar machinery and regulating sugar imports. Interviewees were also aware of the policy priority incoherences – *‘But indeed, on the one hand, sugar is one of Indonesia’s commodities. We, too, have state-owned enterprises related to sugar factories and others. But on the other hand there is also a need to reduce sugar consumption…’* (Government_food and drug_#1).

The framing of sugar by interviewees – and policy actors more widely – from different sectors pointed to political economy dynamics underlying this evident policy tension and revealed certain barriers to sugar policy formation for health. Interviewees commented that those responsible for promoting sugar production and process (i.e. Ministry of Trade, Ministry of Industry and Ministry of Finance) were highly vocal in their beliefs about the important role of sugar in the Indonesian economy and national development. Specifically, they were strong advocates for protecting farmers and business – who rely heavily on sugar production and processing for their livelihoods. Interviewees from the health sector also commented on repeated attempts to regulate sugar consumption being blocked by Ministry of Industry – ‘*I’m telling you this, so whatever happens regarding health regulations that come into contact with industry, economy, it will be brought into the realm of politics…the reality is that it [health regulations] is facing obstacles, blocks from the [Ministry of] industry, so there are those from the [Ministry of] industry who protest to postpone it for a while, that’s the story.’* (Government_health_#1).

#### Alignment between ministries framing sugar as an economic good and ministries framing sugar as a commodity for food security

Policies related to increasing sugar production for national economic development were aligned with policies promoting sugar for food security purposes. Policy alignment appeared between ministries who framed sugar as an economic good (e.g. Ministry of Trade and Ministry of Industry) and ministries who framed sugar as a key commodity for food security (e.g. Ministry of Finance and Ministry of Agriculture). The framing of sugar as an economic good was evident in policies that promoted increased sugar production, backed by historical framings/priorities around sugar as a commodity for food security and economic development. This framing was also evident in policies that supported and protected the interests of sugar farmers and small businesses. Interviewees were aware of this framing, particularly around the support from the Ministry of Trade and Industry for farmers – *‘Ministry of Industry and Trade…these people say that we have to defend farmers, the same with tobacco, we have to defend farmers…’* (Government_health_#1). This powerful alliance between trade and industry demonstrates a form of discursive power by creating discourse around farmer and small business welfare, which impacts the ability of the Ministry of Health and other health advocates to create effective nutrition policy.

## Discussion

### Incoherences, tensions and policy alignment

This study examined politico-economic factors relating to sugar policy in Indonesia, in order to identify strategies to support the improved integration of national food and nutrition security priorities with respect to sugar. The current and historical context of sugar policy in Indonesia, as well as competing policy priorities, have led to *incoherences* between ministries that seek to reduce sugar consumption for health and ministries promoting sugar consumption for food security. Existing policy priority incoherences and barriers to the creation and implementation of sugar policy for health resulted in *tensions* between policies aimed at reducing sugar consumption to combat rising rates of NCD and policies actively promoting sugar production, processing and trade in line with economic development goals. Additionally, a *policy alignment* was observed between ministries that seek to strengthen sugar production and processing for economic development and food security purposes. Indonesia’s history of malnutrition has shaped perceptions around food, sugar and food security – which in turn has influenced the policy landscape. Different forms of power (such as discursive and authoritative) influenced the impact of ministry policies relevant to sugar.

In the context of the rising double burden of malnutrition, many countries still lack policies that adequately address both under and overnutrition, with many focused on increasing calorie availability. The Government of Indonesia is not alone in struggling to balance health, food security and economic development. These findings are consistent with other studies that indicate that pursuing economic growth has made it difficult to regulate the sugar industry to achieve nutrition and health outcomes^(30–32)^. For example, India and Rwanda also face increasing challenges with balancing economic growth and nutrition priorities. Sugar is of economic importance in India, which is the second largest producer of sugarcane globally^(32)^ and is the largest sugar-consuming country globally, accounting for 16 % of total sugar consumption^(32)^. Under the Essential Commodities Act 1995, sugar and sugarcane are considered essential commodities in India and are subsidised^(32)^ – making them cheaper for consumers and businesses to purchase. In Rwanda, growing urbanisation and economic growth have led to changes in dietary patterns, characterised by increased consumption of foods high in salt, fat and sugar^(31)^. Similar to the situation in Indonesia and India, despite the increasing burden of NCD, Rwandan food policies have focused primarily on food production to address food insecurity. At the same time, policies aimed at bolstering the production and processing of sugar have been adopted in Rwanda^(31)^. Addressing the double burden of malnutrition in Indonesia requires an integrated nutrition policy agenda, that balances nutrition and economic priorities.

The incoherences and tensions surrounding sugar policy priorities reflect implicit power differentials between different sectors of government. The analysis indicated that the Ministry of Health was able to exert discursive power, based on their mandate for health and high level of recognition of NCD as a major policy problem. This contributed to the Ministry of Health being able to change the understanding of sugar in the Indonesian diet, particularly through policies linking an overconsumption of sugar to diet-related NCD. Alongside this however, Ministry of Industry and Ministry of Trade were able to exert both discursive and authoritative power, whereby keeping ‘practical’ change on the health side limited, including through vocal opposition to sugar-related regulation (discursive), and also in generating a scale-up of industry and trade sector policies that directly supported and promoted the strengthening of Indonesia’s sugar sector (authoritative). This study highlights the complexity of designing and implementing effective policies to reduce the double burden of malnutrition that is not undermined by competing policy priorities and actions, whilst governments are also pursuing important economic policy goals that include growing the (economic) food sector^(33)^. However, the recognition of policy incoherence and tensions by the interviewees in this study is a possible reason for optimism; understanding where the conflicts and incoherences lie is an important step towards aligning nutrition and economic priorities^(34,35)^.

This study also highlighted the real concern among policy actors in Indonesia for farmers and small businesses, and what more tightly regulated sugar policies for health purposes would mean for their livelihoods – concerns echoed in past tobacco control efforts. Agriculture in Indonesia accounts for 12·4 % of GDP and is the second largest source of employment, particularly in rural areas^(36)^. Sugar processing and exporting also contribute to the economy as well and employ a large number of people. In this context, alongside the Governments vision of achieving self-sufficiency in food production, any policies that would restrict the production, processing and trade of sugar, without support for alternatives, would significantly impact farmer and small business livelihood^(5)^. Experience from tobacco control policies reflects this underlying tension between public health policymakers who seek to improve nutrition and efforts to support cash crops (such as tobacco and sugar) to increase economic growth^(5)^.

### Strengths and limitations of the study

This article presents a theory-informed analysis of the sugar-relevant policy environment in Indonesia. The study was a collaboration between researchers in Australia and Indonesia, and this collaboration included translation of policies from Bahasa to English using translation software, with data checked and confirmed by native co-authors. However, this may have resulted in some misinterpretation of translated policies. The team were successful in obtaining interviews with most key sectors; however, the lack of participation from the Ministry of Agriculture meant that we had to rely only on documentary data and other sectoral perspectives with respect to agriculture sector priorities. A significant strength of the paper was the triangulation of the policies with the interview data. The authors are also aware of new laws and policies that have been adopted after the data collection phase, which may change the analysis.

### Looking to the sweet, sweet future – strategies and implications for improving nutrition-related sugar policy

Balancing nutrition, economic growth and farmer welfare is a challenge for Indonesian policymakers. Improving nutrition-related sugar policy spans multiple government sectors and levels of government. Ensuring that policy priorities related to sugar are coherent with global nutrition guidelines is important for improving nutrition and health-related outcomes in Indonesia.

#### ‘Win-win’ situations: creating policy measures that bridge competing priorities

There were clear competing priorities between government ministries and other stakeholders related to sugar policy. Co-construction of policy mandates that move support away from sugar production, trade and distribution – towards sustainable and healthy consumer products and production practices will help safeguard the livelihoods of farmers and the health of the population. However, acknowledging the concerns around farmer welfare, this shift needs to embody the principles of a ‘just transition’ – a process that secures workers’ rights and livelihoods whilst economies shift to sustainable production^(37,38)^. Guiding principles of a just transition include strong social consensus, policies that respect, promote and realise fundamental rights of workers, tailored policies that reflect a country’s stage of development and economic sectors and fostering international cooperation^(37)^. Embodying a just transition framework within policy development will help ensure the welfare and economic livelihoods of those working within the sugar sector^(38)^. Whilst the Government of Indonesia is in the process of drafting the new National Long-Term Development Plan (Rencana Pembangunan Jangka Panjang Nasional/RPJPN) for 2025–2029, this presents a pivotal period for aligning competing priorities.

#### Opportunities to diversify agricultural production and value-adding industries

Improving diversification out of sugar production, as well as creating greater incentives for farmers to cultivate other crops, will have potential benefits with improved livelihoods, reduced vulnerability to external shocks such as climate change, increased agricultural diversity and environmental sustainability^(5)^. This has already been translated into various policies that highlight the benefits of increased agricultural diversification for farmer income, food price fluctuations and the preservation of natural resources. However, greater intersectoral collaboration between ministries is needed to identify more opportunities to diversify agricultural production and value-adding industries and how to implement and coordinate such policies and initiatives.

#### Who to engage with?

Knowing who to engage with in the policy process to create an effective nutrition policy is a key consideration for any country that seeks to balance vested interests within the public health space. This Indonesian case study provides insight into the range of stakeholders involved in policy related to the production, processing and trade of sugar, as well as nutrition and NCD-relevant policy. Effective nutrition policy formulation requires coordination and collaboration from multiple stakeholders – from relevant government ministries such as health, agriculture and finance, to sugar farmers and farmer associations, sugar processors and small businesses – who have a vested interest in these policy decisions. Public health advocates face increasing challenges of opposition when unhealthy industries are positioned as contributors to economic growth in the eyes of the government^(30)^. However, building relationships with key stakeholders and forming diverse, well-connected coalitions helps protect nutrition policy from vested interests whilst also managing conflicts of interest^(39,40)^.

### Conclusion

The high political priority of sugar as an economic and food security commodity presents a challenge to policymakers seeking to regulate sugar for health and nutrition, in Indonesia and globally. The increasing prevalence of NCD in Indonesia requires thoughtful consideration in creating an effective and meaningful nutrition policy relevant to sugar. Ensuring a just transition to protect the livelihoods of sugar farmers in Indonesia is crucial in the design and implementation of such policies. Creating opportunities to diversify agricultural production, as well as engaging with relevant stakeholders, will help support improved integration of national nutrition and food security priorities with respect to sugar.

## Supporting information

Johnson et al. supplementary material Johnson et al. supplementary material
