# Supplementary material for: Looking towards the sweet, sweet future: a political economy analysis of sugar and nutrition policy in Indonesia
Source: Public Health Nutr. 2025 Jun 17;28(1):e112. doi: 10.1017/S1368980025100566 (PMC12305376; doi:10.1017/S1368980025100566)
Supplement: Johnson et al. supplementary material [file S1368980025100566sup001.docx]

**PANDUAN WAWANCARA MENDALAM**

**INTERVIEW GUIDE**

# **Section 1. About the interviewee (all interviewees)**

1. Dapatkah Anda ceritakan sedikit tentang peran Anda di instansi Anda saat ini?

Could you please tell us a little about your role in your current institution?

1. Bagaimana Anda mendeskripsikan, prioritas lembaga Anda saat ini?

How would you describe the current priorities [of your agency]?

1. Apakah peran institusi Anda dalam bidang Gizi? Bagaimana pembagian tanggung jawab tersebut kepada pemerintah tingkat nasional, provinsi, dan kabupaten?

What are your agency roles on nutrition? How is the responsibility spread across national, provincial and district governments?

*Sekarang saya ingin mengajukan beberapa pertanyaan tentang pemikiran dan pendapat Anda terkait gizi dan kebijakan gizi.*

*I would now like to ask a few questions about your thoughts and opinions related to nutrition and nutrition policy.*

1. Berdasarkan perspektif Anda, Apa masalah gizi utama di Indonesia?

From your perspective, what do you think are the main nutrition problems in Indonesia?

1. Menurut Anda, apakah Pemerintah harus terlibat aktif dalam promosi gizi? Apakah intervensi yang menurut Anda paling efektif?

Do you think that the government should be actively involved in achieving good nutrition? What are the most effective interventions?

Prompts: Semua tipe malnutrisi

Prompts: all forms of malnutrition

1. Menurut pendapat Anda, bagaimana gizi dibandingkan dengan prioritas Pemerintah lainnya, seperti pertumbuhan ekonomi (lebih atau kurang penting?) atau lingkungan, dan lain-lain. Dapatkan Anda memikirkan prioritas lain yang mungkin lebih penting?

In your opinion, how important is nutrition compared to other government priorities, like economic growth (more or less important?) or the environment etc. – can you think of other priorities that are probably more important?

1. Menurut pendapat Anda, pemangku kepentingan manakah yang memiki peran terpenting agar dalam memperbaiki gizi Indonesia?

In your opinion, which stakeholders play a crucial role in achieving good nutrition?

# **Section 2. Nutrition specific policy (nutrition/health experts only)**

*Sekarang, saya akan bertanya tentang pendapat dan persepsi Anda mengenai gizi di Indonesia.*

*Now, I would like to ask about your opinions and perceptions regarding nutrition in Indonesia.*

1. Menurut Anda apa yang dimaksud dengan pola makan sehat?

What is your opinion regarding what healthy diet is?

1. Kami tertarik untuk mempromosikan pola makan sehat untuk meningkatkan kesehatan. Menurut Anda, apa alasan utama mengapa orang tidak selalu mengonsumsi makanan sehat (misalnya, mengapa orang minum banyak minuman manis? Makan makanan yang tinggi natrium?)

We are interested in promoting healthy diets to improve health. What do you think are the main reasons why people don’t always eat healthy diets (e.g. why do people drink so much sugary drink? Eat high sodium food?)

Prompts: pendidikan, ekonomi, budaya, agama, geografi, lingkungan makanan

Prompts: education, economic, culture, religion, geography, food environment

1. Menurut pendapat Anda, siapa orang atau organisasi utama di luar pemerintahan yang memiliki kepentingan dengan kebijakan gizi?

In your opinion, who are the main people or organizations outside of the government which have interests or a role in nutrition policy?

- 1. Diantara pihak yang telah disebutkan, menurut Anda siapakah yang berpengaruh?

Who do you think is influential?

1. Apakah ada koordinasi antar-sektor untuk kebijakan pangan dan gizi di tingkat nasional (horizontal)?
2. Is there multi-sectoral coordination for food and nutrition policy at a national?
   1. Seperti apa bentuk koordinasi tersebut?

What does this look like?

- 1. Bagaimana bentuk koordinasi kebijakan pangan dan gizi di tingkat provinsi dan daerah (vertical)?

How does coordination for food and nutrition policy happen for provincial and local policy?

**Section 3. Food policy priorities (non-health sectors only)**

*Sekarang kami akan membicarkan tentang struktur di dalam pemerintah yang berkaitan dengan kebijakan pangan secara luas, termasuk pertanian, perdagangan dan industry. Kami telah mengidentifikasi berbagai kebijakan pada sektor-sektor ini yang berkaitan dengan pangan.*

*Now we would like to talk about structures within the government related to food policy broadly, including agriculture, commerce, and trade. We have identified many policies across these sectors that related to food.*

1. Siapakah sektor-sektor utama yang terlibat dalam kebijakan terkait pangan?

What is the lead policy sectors for food policy?

1. Menurut Anda, apa prioritas kebijakan pangan utama pemerintah Indonesia?

In your opinion, what are the key food policy priorities for the government of Indonesia?

1. Apakah ada koordinasi antar-sektor untuk kebijakan pangan dan gizi di tingkat nasional (horizontal)?

Is there multi-sectoral coordination for food policy at a national?

- 1. Seperti apa bentuk koordinasi tersebut?

What does this look like?

- 1. Bagaimana bentuk koordinasi kebijakan pangan dan gizi di tingkat provinsi dan daerah (vertical)?

How does coordination for food policy happen for provincial and local policy?

# **Section 4. Attention to nutrition in policy making across sectors (all interviewees)**

*Kami mengetahui bahwa gizi hanyalah satu dari banyak tujuan yang ingin dicapai ketika pengambilan keputusan pembuatan kebijakan terkait pangan, di antara Kementerian Perdagangan, Pertanian, dan Keuangan. Berdasarkan perspektif kesehatan masyarakat, kami berusaha meningkatkan perhatian terhadap gizi dalam pembuatan kebijakan sistem pangan. Secara khusus, untuk meningkatkan akses terhadap makanan sehat bagi masyarakat miskin.*

*We understand that nutrition is only one of many objectives that are taken into account when making policy decisions that related to food, across Ministries of Trade, Agriculture, and Finance. From a public health perspective, we are looking to increase the attention to nutrition in the making of the food system policy. In particular, to increase access to healthy food in poor communities.*

1. Seperti yang Anda ketahui, Indonesia menghadapi tantangan gizi yang berkaitan dengan pola makan kurang gizi dan tidak sehat yang menyebabkan PTM; Menurut Anda, apakah mungkin untuk mengatasi kedua masalah ini melalui kebijakan, pada saat yang bersamaan? Bagaimana? Menurut Anda, apakah kebijakan saat ini dapat mencapai hal ini?

As you may be aware, Indonesia faces nutrition challenges relating to both underweight and unhealthy diets causing NCDs; In your opinion, is it possible to address both of these problems through policy, at the same time? How? Do you think current policies are achieving this?

1. Apakah ada kebijakan yang Anda lihat sebagai “kebijakan tugas ganda”, di mana satu inisiatif kebijakan mempromosikan pola makan sehat untuk mencegah obesitas, dan mengatasi kekurangan berat badan?

Are there any policies that you see as being “double-duty policies”, where a single policy initiative both promotes healthy diets to prevent obesity, and addresses underweight?

# **Section 5: Case Studies**

*Saya sekarang akan bertanya tentang inisiasi kebijakan tertentu.*

*I would now like to ask you about some specific policy initiatives.*

Kami memiliki empat studi kasus mengenai kebijakan, dan kami akan bertanya terlebih dahulu, bagian mana yang Anda paling nyaman untuk membicarakannya: label pangan dan gizi, beban ganda malnutrisi, ASI, dan kebijakan terkait gula.

*We have four case study policies, and would like to ask first, which one you are most comfortable talking about: nutrition labelling, DBM, breastfeeding and sugar policy.*

Case Study 4: Sugar policy

*Kami menyertakan studi kasus kebijakan gula dalam studi kami, karena ini merupakan masalah kesehatan dan ekonomi bagi Indonesia, dan kami ingin memahami bagaimana berbagai kepentingan kebijakan dapat diseimbangkan.*

*We are including a case study of sugar policy in our study, because it is both a health and economic issue for Indonesia, and we would like to understand how diverse policy interests can be balanced.*

1. Menurut Anda, apa visi pemerintah terhadap peran gula dalam perekonomian di masa depan?

In your opinion, what is the governments vision for the future of sugar’s role in the economy?

- 1. Prompts: Apakah mereka ingin menghentikan produksi gula? Apakah mereka berencana memperluas produksi gula dalam negeri?

Prompts: Do they want to phase sugar out? Are they looking at expanding domestic sugar production?

1. Apa saja Kementerian dan Departemen pemerintah yang terkemuka dalam hal kebijakan gula?

What are the leading government Ministries and Departments with respect to sugar policy?

- 1. Apa prioritas mereka terkait kebijakan gula?

What are their priorities, with respect to sugar policy?

- 1. Apa saja tantangan utama dalam penerapan kebijakan [pertanian, keuangan, perdagangan, industri] terkait gula?

What are the main challenges in implementing [agriculture, finance, trade, industry] policy related to sugar?

- 1. Menurut Anda, apakah industri gula memengaruhi kebijakan gula? Jika ya, bagaimana?

Do you think that the sugar industry influences sugar policy? If so, how?

1. Menurut Anda, apakah kesadaran akan gizi dan pola makan sehat berdampak pada kebijakan terkait gula? Mengapa atau mengapa tidak?

In your opinion, has awareness of nutrition and healthy diets impacted on policy related to sugar? Why or Why not?

**Section 6: Opportunities to improve nutrition policy**

*Ini pertanyaan terakhir kami.*

*This is our final question.*

1. Apa yang Anda lihat sebagai peluang untuk mengembangkan struktur tata kelola atau pendekatan konsultasi, untuk memastikan bahwa gizi diperhitungkan dalam penyusunan kebijakan pangan di Indonesia?

In your opinion, what factors would increase the attention given to nutrition (addressing all forms of malnutrition) in policy making, across sectors? For example, institutional structures, skills and capacity of policy makers, or new evidence?

Prompt: Struktur tata kelola atau komite? Bagaimana pemerintah disusun untuk menangani masalah gizi (bagaimana hal ini dapat ditingkatkan)?

Prompt: Governance structure or committees? How is the government structured to address nutrition (how could this be improved)?

Terima kasih. Bisakah Anda memberi tahu kami jika Anda memiliki rekomendasi untuk orang yang dapat berbicara tentang studi kasus lainnya? Atau studi kasus yang sama?

Thank you. Could you please let us know if you have any recommendations for people who can speak to the other case studies? Or the same case study?

Bisakah Anda memberi tahu kami jika Anda memiliki rekomendasi tentang orang-orang yang harus kami wawancarai, di tingkat nasional, provinsi, atau distrik?

Could you please let us know if you have any recommendations for people we should interview, at the national, province or district level?
